# Effect of planting and mowing cover crops as livestock feed on soil quality and pear production

**DOI:** 10.3389/fpls.2022.1105308

**Published:** 2023-01-04

**Authors:** Haoran Fu, Hong Chen, Qingxu Ma, Kefeng Han, Shaofu Wu, Lianghuan Wu

**Affiliations:** ^1^ Zhejiang Provincial Key Laboratory of Agricultural Resources and Environment, College of Environmental and Resource Sciences, Zhejiang University, Hangzhou, China; ^2^ School of Public Affairs, Zhejiang University, Hangzhou, China; ^3^ Shaoxing Grain and Oil Crop Technology Extension Center, Shaoxing, China

**Keywords:** community component, different depth, mowing forage crop, pear yield, soil nutrient content, titratable acids

## Abstract

**Introduction:**

The increasing demand for animal-products has led to an increasing demand for livestock feed. Using cover crop as green manure in orchards is an effective measure to improve fruit yield and quality. However, the effect of mowing cover forage crops as livestock feed on soil quality and crop production is unclear.

**Method:**

Therefore, a 4-year field experiment, which included two treatments, was conducted in pear orchards in Luniao County, China: natural grass (NG) and planting and mowing forage crop ryegrass as livestock feed (MF).

**Results:**

Under MF treatment, most soil nutrient content, especially alkalihydrolysable N (AN), total phosphate (TP), available phosphate (AP), and microbial biomass phosphate (MBP), had decreased significantly (P<0.05), while β-D-glucosidase (BG, C-cycle enzyme) and soil C limitation at 10–20 cm depth and P limitation at subsoil (20–40 cm) was increased. In addition, the soil bacterial community component in topsoil (0–10 cm and 10–20 cm) and fungal community component in topsoil and subsoil were changed in the MF treatment. Network analysis showed that MF treatment had a lower edge number in topsoil but the community edge numbers increased from 12794 in NG to 13676 in MF in subsoil. The average weight degree of the three soil layers in MF treatment were reduced, but the modularity had increased than that in NG. For crop production, MF treatment was 1.39 times higher in pear yield and titratable acids (AC) reduced from 0.19% to 0.13% compared with NG. These changes were more associated with the indicators at the subsoil, especially for TP, AN, pH, and F-NMDS1 (non-metric multidimensional scaling (NMDS) axis 1 of fungi).

**Discussion:**

These results provide data support for the feasibility of planting and mowing forage crops as livestock feed on orchards as well as a new idea for the integration of crop and livestock.

## 1 Introduction

To meet the demand of the affluent population for animal-sourced food, animal products have rapidly increased in the recent decades (Raney et al., 2009). In developed countries, over half of the protein was supplied by animal products and a sharp increase was experienced in developing countries (FAO, 2019). To ensure the supply of these animal products, the number of feedlots is rapidly increasing, which means more forage should be supplied. At the same time, humans are facing a great challenge regarding food supply to ensure food security. It has been determined that the total grain production needs to be increased by 60%–110% to meet the demand in 2050 when compared with the current levels (Tilman et al., 2011). Therefore, it is critical to provide sufficient feed for livestock while not compromising food security. At present, grass is a vital forage for livestock production, accounting for approximately 50% of the total livestock intake (Hasha, 2002; Herrero et al., 2013). However, in China, the world’s largest market for animal products, grassland productivity has dropped significantly in recent years, resulting in the great disparity between the supply and demand of forage across different seasons and regions (Li et al., 2016; Jin et al., 2021). Thus, exploring different grass planting patterns can effectively solve the insufficient feed problem.

Fruit orchard is an important component of the agricultural industry in China, with approximately 1 billion hectares of land area and 32% of the total yield of the world in 2018 (FAO, 2019). However, in most commercial orchards, the inter-canopy area is usually bare soil caused by intensive management of herbicides and soil tillage (Fang et al., 2021). Intercropping cover crops with orchards can supply forage, which is a sustainable orchard management strategy. Compared with bare orchards, natural grass has a positive effect on soil physicochemical properties, microbial activities, and fruit yield and quality (Hoyt and Hargrove, 1986; Wardle et al., 2001; Milgroom et al., 2007). Monteiro and Lopes (2007) demonstrated that natural grass is more conducive to achieve a balanced supply of mineral elements and improve fruit yield and quality. In fact, many forage crops, which can be used as livestock feed, have been grown in orchards as cover crops. For example, ryegrass (*Lolium perenne L.*) was usually treated as a cover crop in fruit orchards (Wang et al., 2020; Piltz et al., 2021). After a cover crop is harvested, it is generally spread on the orchard surface as green manure. The positive effects of the long-term application of green manure on soil quality and fruit production have been reported. Green manure can directly improve soil properties and fertility and indirectly affect the activity and community structure of microorganisms to reduce the occurrence ratio of diseases, and improve yield and quality of fruit trees (Srivastavaak et al., 2007; Gomez-munoz et al., 2014; Wang et al., 2016; Deakin et al., 2018). However, the effect of planting and mowing cover crops as livestock feed on soil quality and fruit production is unclear.

Compared with northern China, the temperature and humidity conditions of pear orchards in southern China are more suitable for rapid growth of forage grass. In addition, pear (Pyrus spp.) is a vital cash crop that is widely cultivated in China. The annual ryegrass is characterized by a high grass yield and strong cold resistance, and is a common winter cover crop in the orchards of southern China (Fu et al., 2021). Thus, this study on pears was undertaken with following objectives: To 1) assess the effect of mowing ryegrass cover crops on soil quality at different depths; 2) quantify the influence of mowing cover crops on pear yield and quality; 3) analyze the correlation between soil quality indicators at different depths and pear production.

## 2 Material and methods

### 2.1 Description of study site

The study site was located at Luniao county, Zhejiang province, China (30°27′N–30°28′N, 119°43′E–119°46′E). This region is characterized by subtropical monsoon climate with a mean annual precipitation and temperature of 1350 mm and 16.0°C, respectively. The main cultivar is “Cuiguan”, with an area of 533.3 ha.

The spacing in the rows and between rows of every 22-year-old tree was 4 m and 3 m, respectively. To analyze the effect of mowing cover crops as livestock feed on soil quality, and pear yield and quality, we set up two treatments since 2018: (1) natural grass (NG); and (2) planting and mowing ryegrass cover crop as livestock feed (MF). The annual ryegrass (*Lolium multiflorum Lam.*) planted in November and mowed three times in March, April, and May after the next year was the same with NG treatment. According to the local smallholders, the total N, P_2_O_5_, and K_2_O fertilization rates in different treatments were the same, with 424.2 kg ha^−1^, 386.4 kg ha^−1^, and 323.4 kg ha^−1^, respectively, and the other management was similar.

### 2.2 Soil sampling and determination of soil properties

Soil sampling was done in July 2021. The soil samples were collected from the same pear trees as the pear samples. The soil was divided into topsoil (0–10 cm, 10–20 cm) and subsoil (20–40 cm), and each soil sample was a mixed sample of five replications. After being mixed evenly, the soil sample was divided into three parts: the first part was stored at −80°C for the analysis of soil bacteria and fungi; the second part was used as fresh soil to measure soil microbial biomass and enzyme activity; and the last one was air-dried to determine the soil physicochemical properties.

Soil pH, soil organic carbon (SOC), total nitrogen (TN), alkalihydrolysable N (AN), total phosphate (TP), available phosphate (AP), and available potassium (AK) were measured using standard methods as described in Bao (2000). Microbial biomass carbon (MBC), microbial biomass nitrogen (MBN), and microbial biomass phosphorus (MBP) were measured using the chloroform fumigation extraction method (Brookes et al., 1985; Vance et al., 1987).

### 2.3 Soil enzyme activities and microbial resource limitation

C acquisition enzyme activities [β-D-glucosidase (BG)], N acquisition enzyme activities [β-Nacetylglucosaminidase (NAG)], and P acquisition enzyme activities [acid phosphatase (ACP)] were determined by the 96-well-microplate protocols German et al. (2011) and the detailed method was described in Chen et al. (2018) and Jing et al. (2017).

Microbial resource limitation was calculated using the vector analysis of ecoenzymatic stoichiometry. The length (L) and vector angle (A°) were calculated according to Moorhead et al. (2016). A relatively longer vector L means a greater C limitation. A larger Vector greater than 45° indicates P limitation, and a lower Vector less than 45° indicates N limitation. The calculation of vector L and Vector A are Equation (1) and (2):


(1) $$\text{Vector L }=\;\sqrt{{\left(\frac{\ln BG}{\ln ACP}\right)}^{2}+{\left(\frac{\ln BG}{\ln NAG}\right)}^{2}}$$

(2) $$\text{Vector A }({}^{\circ})\;=\text{ Degrees }(\text{ATAN}2(\left(\frac{\ln BG}{\ln ACP},\frac{\ln BG}{\ln NAG}\right)))$$

### 2.4 Soil bacterial and fungal community composition

Soil DNA was extracted from approximately 0.5 g of soil with the FastDNA Spin Kit (MP Biomedicals, Solon, OH, USA) according to the manufacturer’s instructions. The spectrophotometer (Thermo Scientific, Waltham, MA, USA) was used to analyze the concentration and quality of the DNA obtained. The 16SrRNA genes in the V4–V5 region were amplified by the primers 515F (GTGCCAGCMGCCGCGGTAA) and 907R (CCGTCAATTCMTTTRAGTTT) and the ITS1 region were amplified using the primers ITS1-F (5′-CTTGGTCATTTAGAGGAAGTAA-3′) and ITS2-2043R (5′-GCTGCGTTCT TCATCG ATGC-3′) (Xiong et al., 2017). The PCR cycle conditions were the same with that of Tang et al. (2022). Sequencing was performed by Illumina NovaSeq platform. Each representative sequence was processed using the UNITE (version 8.0, https://unite.ut.ee) and SILVA (https://www.arb-silva.de/) database. The total bacterial and fungal high-quality sequences were 2,639,562 and 2,637,285 with an average read count per sample of 87,985 (ranging from 84,087 to 91,568) and 87,909 (ranging from 83,869 to 91,799), respectively.

### 2.5 Analysis of pear yield and quality

Fruits were sampled simultaneously with the soil samples. Five healthy pear trees from NG and MF treatment were selected and each tree picked up eight peripheral fruits from different positions (east, south, west, and north). All fruits were taken back to the laboratory for analysis of yield and quality indicators.

Pear yield is obtained by multiplying the number of fruit per tree by the average weight per fruit used to determine fruit quality. The titratable acid (AC) and soluble solids were determined by NaOH neutralization titration method and Abbe refractometer determination method, respectively; 2–6 dichloroindophenol titration and salicylic acid method was used to determine the content of Vitamin C and soluble sugar, according to Zhang et al. (2020).

### 2.6 Data visualization and statistical analysis

Data processing and visualization were performed using R software (version 4.0.3), Microsoft Office Excel 2016 (Microsoft Corporation, Redmond, WA, USA) and SPSS 20.0 (SPSS Inc., Chicago, IL, USA). The differences in soil properties between soil layers, soil treatments, and their interactions were conducted using one-way analysis of variance (ANOVA) using SPSS 20.0. Significant differences were detected using a least significant difference multiple range test with p ≤ 0.05. The difference of soil bacterial and fungal community structure was evaluated by nonmetric multidimensional scaling (NMDS), based on Bray–Curtis dissimilarities. The first 300 fungal operational taxonomic units were selected for co-occurrence network analysis to uncover the bacterial and fungal relationships. The correlations between the different components of the microbiome were derived by the “psych” package in R and correlation coefficients (R) >0.8. BH-adjusted p-values<0.05 were selected to construct the co-occurrence network and Gephi (http://gephi.github.io/) was used to generate network visualization. The relationship between soil quality indicator and pear yield and quality were performed using the “corrplot” package in R and ‘randomForest’ package was carried out to achieve random forest analysis.

## 3 Results

### 3.1 Soil quality

#### 3.1.1 Soil chemical properties

Nutrient content decreased with increasing soil depth, except for soil pH and MBP (**Table 1**). At the same depth, there were no significant differences in TN and MBN between NG and MF treatment (**Table 1**). At all the three depths, the content of AN, TP, AP, and MBP under MF treatment was lower than that of NG (**Table 1**). Compared with NG, the SOC at 10–20 cm had significantly decreased while MBC was significantly increased in MF treatment (**Table 1**). The levels of soil pH at 10–20 cm and 20–40 cm depth in MF treatment were significantly higher (P< 0.05) than those in NG and different depths had no significant effect on soil TN and MBN (**Table 1**).

**Table 1:** Soil chemical characteristics of different soil layers (0–10 cm, 10–20 cm, and 20–40 cm) under different treatments (NG, natural grass; MF, planting ryegrass and mowing as feed).

Indicator	NG			MF		
	0-10cm	10-20cm	20-40cm	0-10cm	10-20cm	20-40cm
pH	5.12 ± 0.33bc	5.04 ± 0.40c	5.07 ± 0.30c	5.45 ± 0.11ab	5.44 ± 0.08ab	5.55 ± 0.14a
SOC	21.49 ± 2.61a	16.14 ± 2.33b	9.80 ± 3.39c	18.50 ± 2.97ab	10.83 ± 1.20c	8.53 ± 2.58c
TN	1.34 ± 0.13a	0.95 ± 0.25bc	0.71 ± 0.16d	1.16 ± 0.20ab	0.79 ± 0.12cd	0.67 ± 0.14d
AN	185.08 ± 27.38a	127.68 ± 8.19b	79.80 ± 13.61c	136.64 ± 10.96b	99.12 ± 15.79c	50.96 ± 5.29d
TP	1.34 ± 0.20a	1.21 ± 0.18a	0.90 ± 0.06b	0.89 ± 0.10b	0.72 ± 0.14bc	0.61 ± 0.10c
AP	100.87 ± 29.75a	89.60 ± 13.81a	50.93 ± 14.40b	53.83 ± 20.34b	32.83 ± 18.68bc	10.50 ± 8.2c
AK	255.00 ± 32.79a	181.00 ± 15.65bc	151.40 ± 32.49bc	205.33 ± 67.47b	172.80 ± 53.87bc	127.2 ± 39.8c
MBC	105.9 ± 9.8a	86.9 ± 8.1b	72.3 ± 12b	104.5 ± 7a	102.3 ± 9.6a	82.5 ± 4.3b
MBN	18.5 ± 11.4a	25.4 ± 11.8a	18.8 ± 11.1a	28.6 ± 10.4a	29.5 ± 24.1a	19.8 ± 6.5a
MBP	78.3 ± 37.3a	100.7 ± 43.3a	36.5 ± 20.2b	36.7 ± 19.8b	37.4 ± 16.5b	10.8 ± 11.4c

Values in the same column following different letters suggest significant differences (p< 0.05). Mean ± standard deviation is presented on different treatments. SOC, soil organic carbon; TN, total nitrogen; TP, total phosphate; AN, alkalihydrolysable N; AP, available phosphate; AK, available potassium; MBC, microbial biomass carbon; MBN, microbial biomass nitrogen; MBP, microbial biomass phosphorus.

#### 3.1.2 Soil enzyme activity

**Figure 1:**
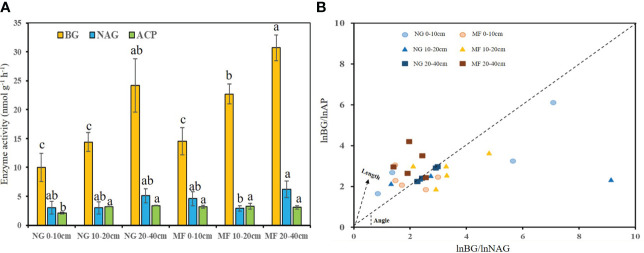
Enzyme activity **(A)** and enzyme stoichiometric constraints **(B)** of different soil layers (0–10 cm, 10–20 cm, and 20–40 cm) under different treatments (NG, natural grass; MF, planting ryegrass and mowing for feed). Changes of vector length (L) and vector angle (A◦) were calculated according to the ratios of the log transformed BG, NAG, and AP (Moorhead et al., 2016). Longer vector L indicates greater C limitation. A vector angle of < 45◦ denotes N limitation, angles > 45◦ denote P limitation. Lowercase letters a, b and c indicate a significant difference (p < 0.05). BG, β-D-glucosidase; NAG, β-Nacetylglucosaminidase; ACP, acid phosphatase.

The activities of BG at 10–20 cm and ACP at 0–10 cm in MF were 1.6 and 1.5 times higher (P< 0.05) than that in NG, respectively, and no significant difference was observed on NAG (**Figure 1**). For stoichiometric constraint, MF treatments showed a stronger C limitation at 10–20 cm, with a longer vector L, and P limitation at 20–40 cm, as demonstrated by a vector angle >45°, compared to NG (**Figure 1**).

#### 3.1.3 Soil microbial community diversity and composition

**Figure 2:**
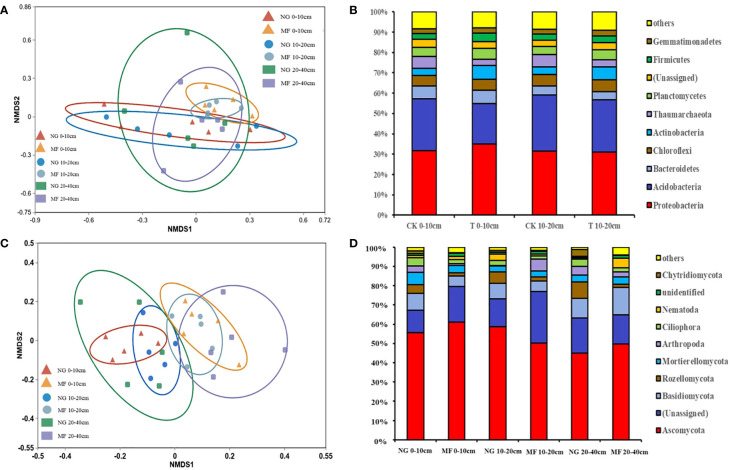
Non-metric multidimensional scaling (NMDS) based on Bray–Curtis dissimilarities **(A, C)** and relative abundance **(B, D)** of bacteria and fungi communities in different soil layers (0–10 cm, 10–20 cm, and 20–40 cm) of different treatments at phylum level. NG, natural grass; MF, planting ryegrass and mowing as feed. SOC, soil organic carbon; TN, total nitrogen; TP, total phosphate; AN, alkalihydrolysable N; AP, available phosphate; AK, available potassium; MBC, microbial biomass carbon; MBN, microbial biomass nitrogen; MBP, microbial biomass phosphorus.

The different treatments had no significant effects on soil bacterial and fungi richness and diversity, despite an increasing tendency (Chao1 and Shannon; **Table S1**). However, the microbial community structure was differed between NG and MF. The results of NMDS based on Bray–Curtis dissimilarities suggested that in different treatments soil bacterial community structure of 0–10 cm and 10–20 cm and fungi community structure of 0–10 cm, 10–20 cm, and 20–40 cm were separated (**Figures 2A, C**).

The changes in the relative abundance of soil bacterial communities at phylum level in the 0–10 cm and 10–20 cm soil layers were similar (**Figure 2A**). Majority of the soil bacterial community is made up of *Proteobacteria* phyla, which accounts for 30.0% on average, with no significant differences from other groups (**Figure 2B**). The relative abundance (RA) of *Acidobacteria* was decreased while *Actinobacteria* was increased in 0–10 cm and 10–20 cm soil layers under MF treatment compared with NG (**Figure 2B**). For fungi composition, the dominant phyla included *Ascomycota*, *Basidiomycota*, *Rozellomycota, Mortierellomycota, and Chytridiomycota*, accounting for more than 70% of all fungi communities (**Figure 2D**). MF decreased the RA of *Basidiomycota* and *Mortierellomycota* at 0–10 cm but increased that of *Ascomycota* (**Figure 2D**). For the 20–40 cm soil layer, the abundance of above fungi increased in the MF treatment (**Figure 2D**). Compared with NG, the RA of *Chytridiomycota* was decreased at 0–10 cm and 10–20 cm depths and that at 20–40 cm depth was increased in MF (**Figure 2D**).

#### 3.1.4 Soil microbial network stability

**Figure 3:**
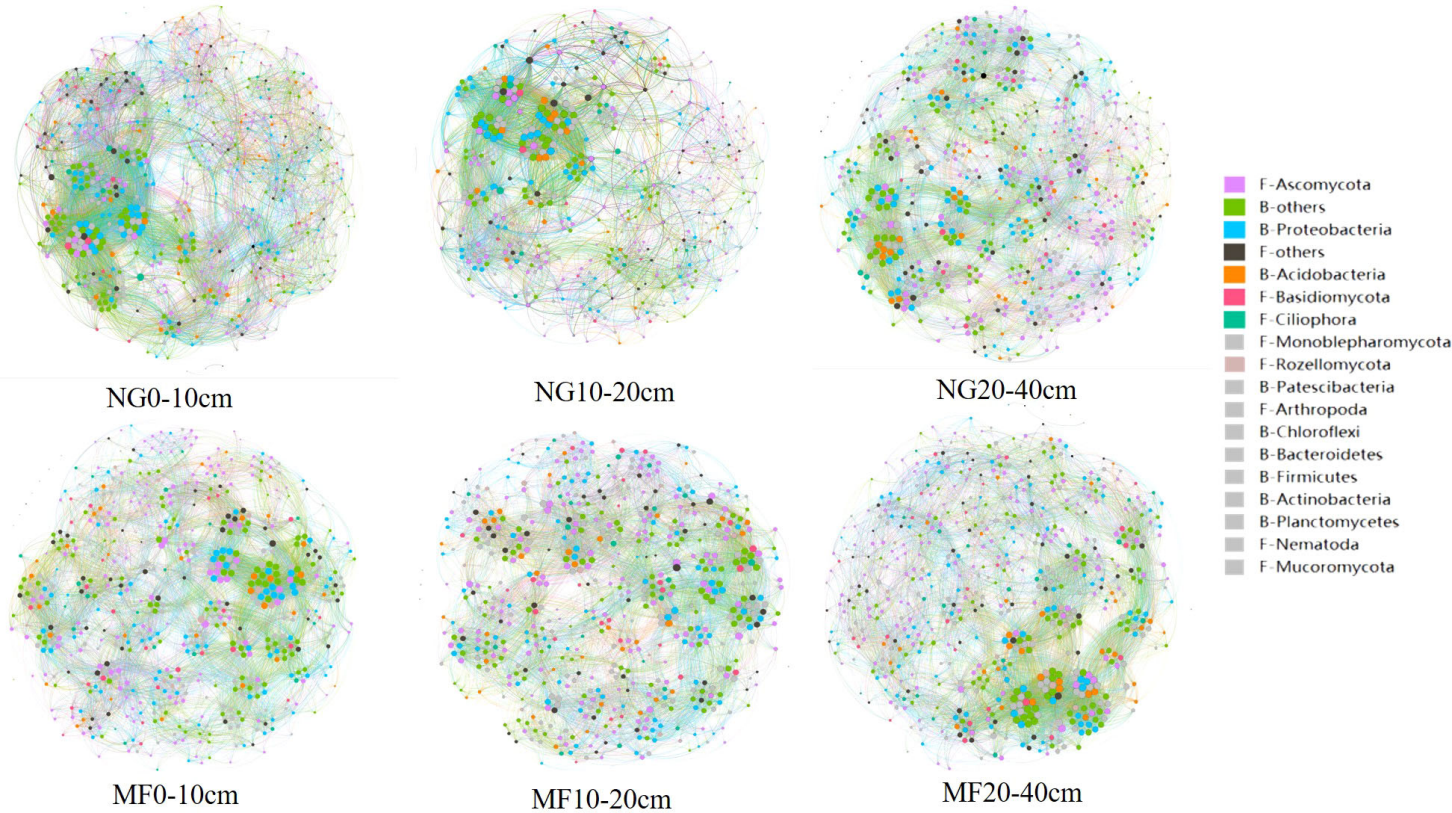
Soil bacterial and fungal co-occurrence networks between NG and MF at different depths based on Spearman’s correlation coefficient (r) at the phylum level (|r| > 0.6, P-value<0.05). Node size is proportional to the connectivity (degree) of each phylum and the different colors indicate different phylum.

Network analysis results showed that MF had a lower edge number in 0–10 cm and 10–20 cm depth compared with NG, in which the edge numbers were 13184 and 12381, respectively (**Figure 3** and **Table S2**). However, community edge numbers increased from 12794 in NG to 13676 in MF at 20–40 cm. In addition, the average weight degree of the three soil layers in the MF treatment were reduced, but the modularity was increased.

The positive and negative links between bacteria and bacteria (B-B), fungi and fungi (F-F), and bacteria and fungi (B-F) at the phylum level were analyzed (**Table 2**). In the three soil layers, the total proportion of positive links in MF was decreased compared with that of NG. For the different soil depths, the proportion of positive correlation on F-F increased, and that of negative correlation decreased at 0–10 cm depth; at 10–20 cm depth, the total link and negative link of F-F increased while that of B-B decreased; the negative link of B-B and B-F was increased while the positive link of F-F and B-F was decreased at 20–40 cm depth.

**Table 2:** Numbers of links in the networks of B-B (Bacterial-Bacterial), B-F (Bacterial-Fungi), and F-F (Fungi-Fungi) obtained for soil samples under different treatments (NG, natural grass; MF, planting ryegrass and mowing as feed) at different depths (0–10 cm,10–20 cm, and 20–40 cm).

	NG0-10cm	NG10-20cm	NG20-40cm	MF0-10cm	MF10-20cm	MF20-40cm
Total links	14566	16200	12794	13814	12831	13676
Positive link	54.1	56.4	62.2	52.3	54.5	53.8
Negative link	45.9	43.6	27.8	47.7	45.5	46.2
B-B						
Positive link	2710 (18.6%)	3340 (20.6%)	2484 (19.4%)	2504 (18.1%)	2438 (19.0%)	2616 (19.1%)
Negative link	2228 (15.3%)	2502 (15.4%)	1250 (9.7%)	2033 (14.7%)	1126 (8.8%)	1930 (14.1%)
F-F						
Positive link	1117 (7.7%)	2201 (13.6%)	1976 (15.4%)	1562 (11.3%)	1821 (14.2%)	1549 (11.3%)
Negative link	1902 (13.1%)	1118 (6.9%)	1129 (8.8%)	1361 (9.9%)	1297 (10.1%)	1343 (9.8%)
B-F						
Positive link	3266 (22.4%)	3490 (21.5%)	3493 (27.3%)	3155 (22.8%)	2731 (21.3%)	3197 (23.4%)
Negative link	3343 (23.0%)	3449 (21.3%)	2462 (19.2%)	3199 (23.2%)	3418 (26.6%)	3041 (22.2%)

### 3.2 Pear yield and quality

Different practices had a significant effect on pear yield and quality. Pear yield under MF treatment was significantly higher than that under NG (1.39 times; **Table 3**). For pear quality, the content of AC was significantly decreased from 0.19% to 0.13% while there were no differences in the amount of soluble solids, Vitamin C, and soluble sugars (**Table 1**).

**Table 3:** Effect of different cultivation modes on pear yield and quality.

	Yield (t ha^-1^)	Soluble solids (%)	Titratable acids (%)	Vitamin C (mg/100g)	Soluble sugars (%)
NG	7.40 ± 1.31a	11.33 ± 0.65a	0.19 ± 0.05a	4.61 ± 2.96a	6.11 ± 1.06a
MF	10.30 ± 1.06b	11.21 ± 0.60a	0.13 ± 0.06b	4.07 ± 2.17a	6.18 ± 1.26a

Values are mean ± standard deviation from 5 replicates. Lowercase letters a and b indicate a significant difference (p< 0.05). NG, natural grass; T, planting ryegrass and mowing as feed.

### 3.3 Correlation of soil quality indicators, pear yield and quality

**Figure 4:**
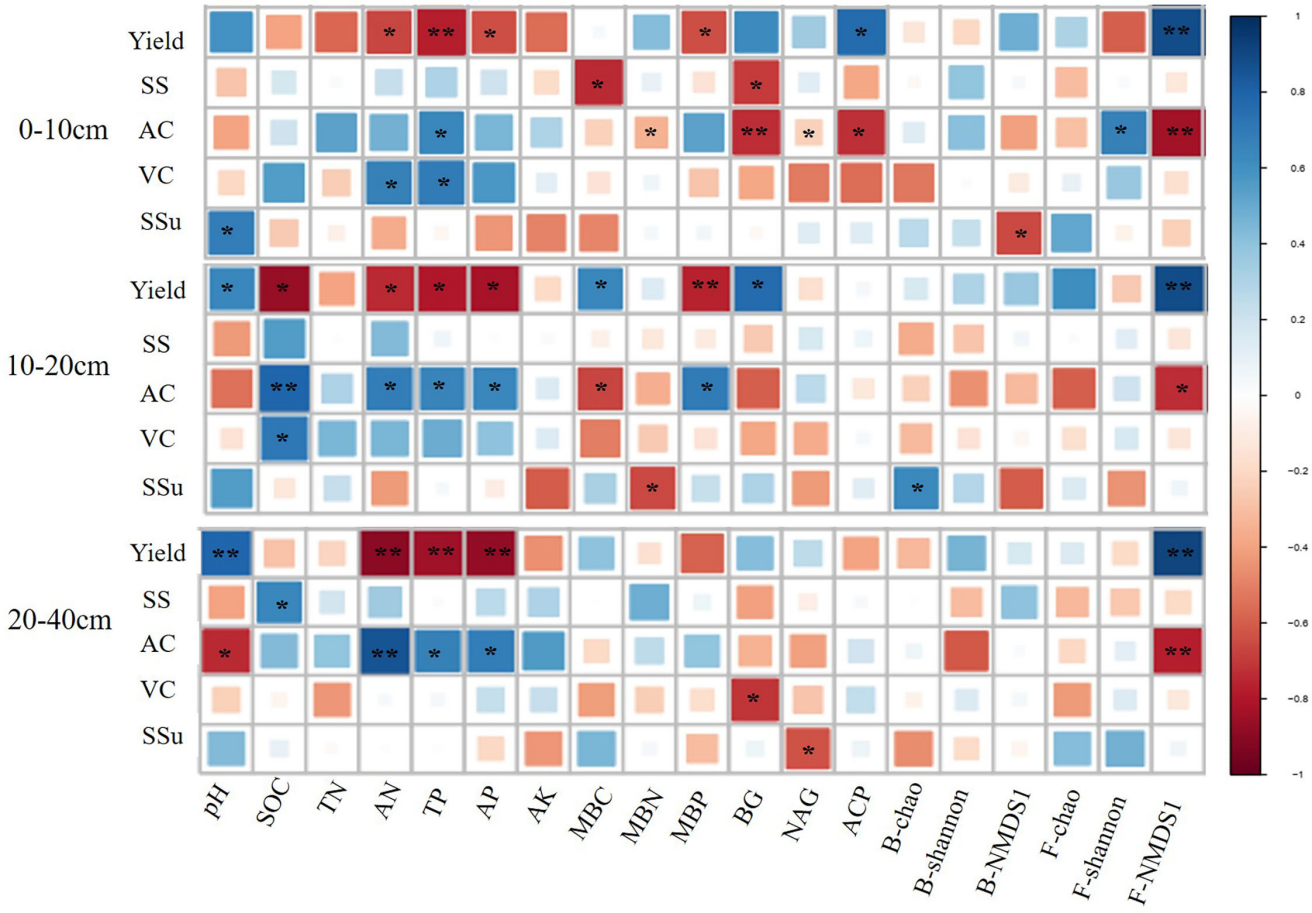
Relationship between soil quality indicators, pear yield and quality at different depths. * and ** represent the significant at 0.05 and 0.01 level, respectively. SOC, soil organic carbon; TN, total nitrogen; TP, total phosphate; AN, alkalihydrolysable N; AP, available phosphate; AK, available potassium; MBC, microbial biomass carbon; MBN, microbial biomass nitrogen; MBP, microbial biomass phosphorus. BG, β-D-glucosidase; NAG, β-Nacetylglucosaminidase; ACP, acid phosphatase; B-chao, bacterial chao index; B-shannon, bacterial Shannon index; B-NMDS1, non-metric multidimensional scaling (NMDS) axis 1 values of the bacterial community; F-chao, fungal chao index; F-shannon, fungal shannon index; F-NMDS1, non-metric multidimensional scaling (NMDS) axis 1 values of the fungal community; SS, Soluble solids; AC, Titratable acids; VC, Vitamin C; SSu, Soluble sugars.

At all the three soil depths, AN, TP, and AP had a positive effect on yield, while F-NMDS1 showed a negative effect (**Figure 4**). In addition, F-NMDS1 positively affected AC at the three depths and AN, TP, and AP had negative effects on AC at 10–20 cm and 20-40 cm soil layers (**Figure 4**).

The random forest models were used to assess the percentage of the total explained variance and the importance of different variables to the change in yield and AC (**Table 4**). The results indicated that the relevant indicators in the 20–40 cm soil layer had the highest percentage of explained variance, with 87.4% and 58.8%. The important factors affecting the yield were TP, F-NMDS1, AN, and AP. The AC depended on AN, pH, TP, and F-NMDS1, and AN was the main factor affecting pear quality.

**Table 4:** Top four importance (percentage of increase in mean square error, %IncMSE) of variables and the percentage of the explained variance (Varex) to the change in yield and AC using the random forest models.

Depth	Rank	Yield			AC		
		Variable	%IncMSE	Var_ex_(%)	Variable	%IncMSE	Var_ex_(%)
0-10cm	1	F-NMDS1	7.7	24.8	BG	6.8	38.4
	2	ACP	5.2		F-NMDS1	6.6	
	3	TP	5.1		TP	4.7	
	4	AN	4.7		ACP	4.0	
10-20cm	1	AP	6.3	80.6	MBC	7.4	46.6
	2	AN	6.1		SOC	6.5	
	3	SOC	5.8		MBP	6.1	
	4	BG	5.8		AN	3.6	
20-40cm	1	TP	8.0	87.4	AN	10.2	58.8
	2	F-NMDS1	7.8		pH	7.3	
	3	AN	7.6		TP	4.7	
	4	AP	7.2		F-NMDS1	4.2	

SOC, soil organic carbon; TP, total phosphate; AN, alkalihydrolysable N; AP, available phosphate; MBC, microbial biomass carbon; MBP, microbial biomass phosphorus. BG, β-D-glucosidase; ACP, acid phosphatase; F-NMDS1, non-metric multidimensional scaling (NMDS) axis 1 values of the fungal community; SS, Soluble solids; AC, Titratable acids; VC, Vitamin C; SSu, Soluble sugars.

## 4 Discussion

### 4.1 Effect of mowing forage crop on soil quality

With the gradual increase in soil depth, the content of most soil nutrients gradually decreased, possibly as a result of fertilization management, which is basically applied to the ground surface. At the same depth, planting ryegrass can significantly increase pH in acidic soil, which similar to the findings of Zhao et al. (2022); MF treatment significantly reduced the contents of AN, TP, and AP in all soil layers, which was mainly related to the fact that ryegrass absorbs nutrients from the soil during the growth process (Liu et al., 2013). At 10–20 cm, SOC of MF treatment soil significantly decreased, but MBC increased significantly, which may be because root exudates stimulate the activity of microorganisms and promote the uptake of carbon by microorganisms.

Soil enzyme activity is an important indicator of soil nutrients cycling (Nannipieri et al., 2012; Hussain et al., 2021). MF increased soil BG, which is consistent with the results of Fernandez et al. (2016). Because BG is primarily responsible for the degradation of macromolecular compounds in plant residues (Zheng et al., 2018), the significant increase in BG in the 10–20 cm soil layer may be related to the large distribution of ryegrass roots. Chitin is mainly generated from fungal cell walls (Zheng et al., 2018), and NAG enzymes are primarily used to degrade chitin to promote nitrogen bioavailability. The insignificant differences in fungal abundance and diversity between different treatments may be an important reason for the lack of significant differences in NAG enzymes. From the perspective of enzyme stoichiometry, the MF treatment showed a greater C limitation in the 10–20 cm soil layer and a greater P limitation in the 20–40 cm soil layer which was mainly associated with the significant decrease in SOM and soil P content, respectively. Therefore, when planting ryegrass in pear orchards and mowing them as feed, measures should be taken to increase SOM in topsoil, and ensure the supply of soil phosphorus in subsoil.

Different treatments had no significant effects on soil microbial richness and diversity, but the bacterial community composition in 0–10 cm and 10–20 cm and fungi in 0–10 cm, 10–20 cm, and 20–40 cm changed greatly under different treatments. Under MF treatment, the greater abundance of *Acidobacteria* under natural grass maybe related to the diversity of plant species under this condition (Foesel et al., 2013), and the increase in abundance in *Actinobacteria* was mainly because microorganisms could only use stubborn carbon sources as carbon source as soil organic C content was decreased in MF (Mbuthia et al., 2015). For fungi community composition, *Ascomycota* and *Basidiomycota* were the most prevalent phyla, which is consistent with previous research on agricultural soil (Feng et al., 2015; Ding et al., 2017). *Ascomycota* is the most ubiquitous phylum, which can decompose the organic substrate (Ye et al., 2020) and *Basidiomycota* can produce massive fruiting bodies and cause decomposition of litter (Frac et al., 2021). *Mortierellomycota*, which plays an important role in the carbon cycle and decomposition of organic matter (Muneer et al., 2021), was found in the different treatments. These fungi play a significant role in storing mineral nutrients, metabolites, and water (Ozimek and Hanaka, 2021), and the increase of RA at 20–40 cm could be very advantageous to plants. The *Chytridiomycota* phylum acts as a bio-converter and decomposer and is a vital component in modern ecosystems (Gleason et al., 2005). The change of this fungi may have had a significant impact on ecosystem functioning.

MF treatment had more modularity, which means it is more stable compared with NG (Deng et al., 2012). Network analysis showed that the positive links were decreased in all the three depths, which indicated that MF reduces microbial cooperation and increases competition. Compared with NG, more F-F positive links and less F-F negative links existed in the 0–10 cm depth in MF treatment, which may be because the carbon source of ryegrass treatment is single, and the fungal microorganisms cooperate to use the carbon source when decomposing. For 10–20 cm, the links of F-F were increased and that of B-B were decreased in MF, which may be related to the significant reduction of SOM in the 10–20 cm soil layer. Bacteria are generally sensitive to small molecules and readily available carbon sources, while fungi are good at utilizing carbon sources in refractory organic matter (Caesar-TonThat et al., 2010; Zhong et al., 2018). In the 20–40 cm soil layer, competition (negative links reducing and positive links increasing) among F-F, B-F, and B-B increased may be due to limited resource conditions.

### 4.2 Change of pear yield and quality

Cover cropping has been used as an important and effective method to improve fruit yield and quality (Fang et al., 2021). Many studies have reported that it can improve soil physical structure (Garcia et al., 2018) and enhance nutrient status (Sanchez et al., 2007; Wei et al., 2017). In the subtropics and temperate or humid zones, ground cover management had a greater benefit on fruit yield, possibly due to the formation of abundant cover crop biomass because of adequate precipitation (Fang et al., 2021). However, there is little information about the effect of mowing forage cover crop on yield and quality. Our results showed that mowing cover crop can improve pear yield and reduce AC compared with natural grass. Many studies have reported that excessive fertilizer application to pear orchards results in high soil nutrition content and negative impact on pear yield and quality, particularly in China (Fu et al., 2021; Wang et al., 2021). The results in southern China indicated that the suitable range of soil AP for sand pears is 10–40 mg kg^-1^, while our research found that the nutrient content under natural grass conditions is more than 50 mg kg^-1^ (Li et al., 2008; **Table 1**). The negative effects on pears may have resulted from too much soil nutrition content, but mowing cover crops removes some of the nutrition and reduces the soil nutrient content into a suitable range. The improvement of soil conditions may enhance fruit tree growth, resulting in higher yields. In addition, Qiu (2021) indicated that the suitable soil pH for the southern pears of China ranges from 5.6 to 7.2 and our results proved that an increase in pH can directly improve pear yield (**Figure 4**). At the same time, the result that ground cover management can significantly reduce fruit acidity to improve fruit quality is similar to that reported by Fang et al. (2021).

The random forest models showed that yield and AC were more associated with the relevant indicators in the subsoil (20–40 cm) rather than those in the topsoil (0–10 cm and 10–20 cm). The results of Zhang (1997) indicated that more than 70% of the roots of pear trees are mainly distributed below 20 cm, so the change of soil physicochemical properties in the subsoil layer has greater impact on pear production. Kuhn and Pedersen (2009) suggested that higher yields and quality under cover crops maybe related to suitable soil concentrations. Therefore, a decrease in AN, TP, and AP contribute to the improvement of yield and quality. In addition, F-NMDS1 has great impact on yield and quality, similar to the results of Tang et al. (2023). In general, under the condition of planting ryegrass and mowing, it is necessary to pay more attention to the changes in soil properties of the subsoil.

## 5 Conclusions

The results indicated that MF reduces the soil nutrient content of AN, TP, AP, and MBP at different depths, increases BG at 10–20 cm depth, and changes enzyme stoichiometric ratio (increasing the C limitation in the 10–20 cm and P limitation in the 20–40 cm layer) compared with NG. In addition, soil bacterial community component in topsoil, fungal community component in topsoil and subsoil, and the soil microbial stability were changed under MF treatment. For crop production, MF treatment showed higher pear yield and lower AC and these indicators were more related to subsoil properties (TP, AN, pH, and FNMDS1). This study revealed mowing cover cops as livestock feed can optimised soil quality and pear production especially for high soil fertility soil caused by excessive fertilization and future studies should focus on subsoil management, especially for TP and AN after planting and mowing ryegrass.

## Data availability statement

The raw data presented in the study are deposited in the NCBI repository, accession numbers PRJNA910514 and PRJNA910501.

## Author contributions

HF: Formal analysis, Writing - original draft. HC: Formal analysis. QM: Writing - review & editing. KH: Formal analysis. SW: Conceptualization. LW: Conceptualization. All authors contributed to the article and approved the submitted version.

## References

[B1] Bao S. D. (2000). Soil and agricultural chemistry analysis (Beijing: Agriculture Publication), 355–356.

[B2] Brookes P. C. Landman A. Pruden G. Jenkinson D. S . (1985). Chloroform fumigation and the release of soil nitrogen: a rapid direct extraction method for measuring microbial biomass nitrogen in soil. Soil Biol. Biochem. 17, 837–884. doi: 10.1016/0038-0717(85)90144-0

[B3] Caesar-TonThat T. Lenssen A. W. Caesar A. J. Sainju U. M. Gaskin J. F. (2010). Effects of tillage on microbial populations associated to soil aggregation in dryland spring wheat system. Eur. J. Soil Biol. 46, 119–127. doi: 10.1016/j.ejsobi.2009.12.004

[B4] Chen X. Ding Z. Tang M. Zhu B. (2018). Greater variations of rhizosphere effects within mycorrhizal group than between mycorrhizal group in a temperate forest. Soil Biol. Biochem. 126, 237–246. doi: 10.1016/j.soilbio.2018.08.026

[B5] Deakin G. Tillston E. L. Bennett J. Passey T. Harrison N. Fernandez F. . (2018). Spatial structuring of soil microbial communities in commercial apple orchards. Appl. Soil Ecol. 130, 1–12. doi: 10.1016/j.apsoil.2018.05.015 30177867 PMC6102658

[B6] Deng Y. Jiang Y. H. Yang Y. F. He Z. L. Luo F. Zhou J. Z. . (2012). Molecular ecological network analyses. BMC Bioinform. 13, 113. doi: 10.1186/1471-2105-13-113 PMC3428680 22646978

[B7] Ding J. Jiang X. Guan D. Zhao B. Ma M. Zhou B. . (2017). Influence of inorganic fertilizer and organic manure application on fungal communities in a long-term field experiment of Chinese mollisols. Appl. Soil Ecol. 111, 114–122. doi: 10.1016/j.apsoil.2016.12.003

[B8] Fang L. F. Shi X. J. Zhang Y. Yang Y. H. Zhang X. L. Wang X. Z. . (2021). The effects of ground cover management on fruit yield and quality: a meta-analysis. Arch. Agron. Soil Sci. 40, 1937607. doi: 10.1080/03650340.2021.1937607

[B9] FAO (Food and Agriculture Organization of the United Nations) (2019). FAOSTAT. database-resources (Rome: Food and Agriculture Organization of the United Nations). Available at: http://www.fao.org/faostat/zh/#home.

[B10] Feng Y. Yu Y. Tang H. Zu Q. Zhu J. Lin X. (2015). The contrasting responses of soil microorganisms in two rice cultivars to elevated ground-level ozone. Environ. Pollut. 197, 195–202. doi: 10.1016/j.envpol.2014.11.032 25576991

[B11] Foesel B. U. Nageie V. Naethex A. Wuest P. K. Weinert J. Bonkowski M. . (2013). Determinants of Acidobacteria activity inferred from the relative abundances of 16S rRNA transcripts in German grassland and forest soils. Environ. Microbiol. 16 (3), 658–675 doi: 10.1111/1462-2920.12162 23802854

[B12] Fernandez A. L. Sheaffer C. C. Wyse D. L. Staley C. Gould T. J. Sadowsky M. J. (2016). Associations between soil bacterial community structure and nutrient cycling functions in long-term organic farm soils following cover crop and organic fertilizer amendment. Sci. Total Environ. 566-567, 949–959. doi: 10.1016/j.scitotenv.2016.05.073 27288977

[B13] Frac M. Pertile G. Panek J. Gryta A. Oszust K. Lipiec J. . (2021). Mycobiome composition and diversity under the long-term application of spent mushroom substrate and chicken manure. Agronomy 11, 410. doi: 10.3390/agronomy11030410

[B14] Fu H. R. Ma Q. X. Ma Z. B. Hu Y. Z. Liu F. Chen K. J. . (2021). Quantifying key internal and external yield-limiting factors for Chinese pear in smallholder dominant areas. HortScience 56 (11), 1395–1401. doi: 10.21273/HORTSCI16115-21

[B15] Garcia L. Celette F. Gary C. Ripoche A. Hector V. G. Metay A. (2018). Management of service crops for the provision of ecosystem services in vineyards: a review. Agric. Ecosyst. Environ. 251, 158–170. doi: 10.1016/j.agee.2017.09.030

[B16] German D. P. Weintraub M. N. Grandy A. S. Lauber C. L. Rinkes Z. L. Allison S. D. (2011). Optimization of hydrolytic and oxidative enzyme methods for ecosystem studies. Soil Biol. Biochem. 43, 1387–1397. doi: 10.1016/j.soilbio.2011.03.017

[B17] Gleason F. H. Letcher P. M. Commandeur Z. Jeong C. E. McGee P. A. (2005). The growth response of some chytridiomycota to temperatures commonly observed in the soil. Mycol. Res. 109, 717–722. doi: 10.1017/S0953756204002163 16080394

[B18] Gomez-munoz B. Hatch D. J. Boi R. Garcia-ruiz R. (2014). Nutrient dynamics during decomposition of the residues from a sown legume or ruderal plant cover in an olive oil orchard. Agric. Ecosyst. Environ. 184, 115–123. doi: 10.1016/j.agee.2013.11.020

[B19] Hasha G. (2002). Livestock feeding and feed imports in the European union: A decade of change (Washington: US Department of Agriculture, Economic Research Service).

[B20] Herrero M. Havlik P. Valin H. Notenbaert A. Rufino M. C. Thornton P. K . (2013). Biomass use, production, feed efficiencies, and greenhouse gas emissions from global livestock systems. Proc. Natl. Acad. Sci. U.S.A. 110, 20888–20893. doi: 10.1073/pnas.1308149110 24344273 PMC3876224

[B21] Hoyt G. D. Hargrove W. L. (1986). Legume cover crops for improving crop and soil management in the southern united states. Hortscience 21 (3), 397–402. doi: 10.21273/HORTSCI.21.3.397

[B22] Hussain S. Shafiq I. Skalicky M. Brestic M. Rastogi A. Mumtaz M. . (2021). Titanium application increases phosphorus uptake through changes in auxin content and later root formation in soybean. Front. Plant Sci. 12, 743618. doi: 10.3389/fpls.2021.743618 34858450 PMC8631872

[B23] Jin S. Q. Bin Zhang B. Wu B. Han D. M. Hu Y. Ren C. C. . (2021). Decoupling livestock and crop production at the household level in China. Nat. Sustain. 4, 48–55. doi: 10.1038/s41893-020-00596-0

[B24] Jing X. Chen X. Tang M. Ding Z. J. Jiang L. Li P. . (2017). Nitrogen deposition has minor effect on soil extracellular enzyme activities in six chinese forests. Sci. Total Environ. 607, 806–815. doi: 10.1016/j.scitotenv.2017.07.060 28711842

[B25] Kuhn B. F. Pedersen L. H. (2009). Cover crop and mulching effects on yield and fruit quality in unsprayed organic apple production. Eur. J. Hortic. Sci. 74, 247–253.

[B26] Liu J. Khalaf R. Ulén B. Bergkvist G. (2013). Potential phosphorus release from catch crop shoots and roots after freezing-thawing. Plant Soil 371, 543–557. doi: 10.1007/s11104-013-1716-y

[B27] Li Z. Wu W. Liu X. (2016). Land use/cover change and regional climate change in an arid grassland ecosystem of inner Mongolia, China. Ecol. Model. 353, 86–94. doi: 10.1016/j.ecolmodel.2016.07.019

[B28] Li M. G. Xie W. L. Xie Z. C. Shi Q. Li J. (2008). Study on the suitable value of mineral nutrition of early-ripening sand pear. J. Fruit Trees 4, 473–477.

[B29] Mbuthia L. W. Acosta-Martínez V. DeBruyn J. Schaeffer S. Tyler D. Odoi E. . (2015). Long term tillage cover crop and fertilization effects on microbial community structure activity: Implications for soil quality. Soil Biol. Biochem. 89, 24–34. doi: 10.1016/j.soilbio.2015.06.016

[B30] Milgroom J. Auxiliadora S. M. Garrido J. M. (2007). The influence of a shift from conventional to organic live farming on soil management and erosion risk in southern Spain. Renewable Agric. Food Syst. 22 (1), 1–10. doi: 10.1017/S1742170507001500

[B31] Monteiro A. Lopes C. M. (2007). Influence of cover crop on water use and performance of vineyard in Mediterranean Portugal. Agric. Ecosyst. Environ. 121, 336–342. doi: 10.1016/j.agee.2006.11.016

[B32] Moorhead D. L. Sinsabaugh R. L. Hill B. H. Weintraub M. N. (2016). Vector analysis of ecoenzyme activities reveal constraints on coupled C, N and P dynamics. Soil Biol. Biochem. 93, 1–7. doi: 10.1016/j.soilbio.2015.10.019

[B33] Muneer M. A. Huang X. M. Hou W. Zhang Y. D. Cai Y. Y. Munir M. Z. . (2021). Response of fungal diversity, community composition, and functions to nutrients management in red soil. J. Fungi 7, 554. doi: 10.3390/jof7070554 PMC8307627 34356933

[B34] Nannipieri P. Giagnoni L. Renella G. Puglisi E. Ceccanti B. Masciandaro G. . (2012). Soil enzymology: classical and molecular approaches. Biol. Fert. Soils 48, 743–762. doi: 10.1007/s00374-012-0723-0

[B35] Ozimek E. Hanaka A. (2021). Mortierella species as the plant growth-promoting fungi present in the agricultural soils. Agriculture 11 (1), 7. doi: 10.3390/agriculture11010007

[B36] Piltz J. W. Rodham C. Wilkins J. F. Hackney B. F. Brown C. G. (2021). Economic returns from cereal and Cereal/Vetch forage crops grown as fodder conservation options for beef and sheepmeat production. Agriculture 11 (7), 664–664. doi: 10.3390/agriculture11070664

[B37] Qiu Y. L. (2021). Effects of three-year fertilization on nitrogen, phosphorus, potassium, yield and quality of soil and fruit in pear orchard. (Chongqing: Master's thesis in Southwest University, (in Chinese)). 31.

[B38] Raney T. Steinfeld H. Skoet J. (2009). The state of food and agriculture 2009: livestock in the balance (Rome, Italy: Food and Agriculture Organization of the United Nations).

[B39] Sanchez E. E. Giayetto A. Cichón L. Fernández D. Aruani M. C. Curetti M. (2007). Cover crops influence soil properties and tree performance in an organic apple (Mmalus domestica borkh) orchard in northern Patagonia. Plant Soil. 292 (1– 2), 193–203. doi: 10.1007/s11104-007-9215-7

[B40] Srivastavaak R. H. Ram L. Singh S. (2007). Yield prediction in intercropped versus monocropped citrus orchards Scientia. Hortic. 114 (1), 67–70. doi: 10.1016/j.scienta.2007.05.005

[B41] Tang S. Zhou J. J. Pan W. K. Sun T. Liu M. J. Tang R. . (2023). Effects of combined application of nitrogen, phosphorus, and potassium fertilizers on tea (Camellia sinensis) growth and fungal community. Appl. Soil Ecol. 181, 104661. doi: 10.1016/j.apsoil.2022.104661

[B42] Tang S. Zhou J. J. Pan W. K. Tang R. Ma Q. X. Xu M. . (2022). Impact of n application rate on tea (Camellia sinensis) growth and soil bacterial and fungi communities. Plant Soil 475, 343–359. doi: 10.1007/s11104-022-05372-x

[B43] Tilman D. Balzer C. Hill J. Befort B. L. (2011). Global food demand and the sustainable intensification of agriculture. Proc. Natl. Acad. Sci. U.S.A. 108, 20260–20264. doi: 10.1073/pnas.1116437108 22106295 PMC3250154

[B44] Vance E. D. Brookes P. C. Jenkinson D. S. (1987). An extraction method for measuring soil microbial biomass c. Soil Biol. Biochem. 19, 703–707. doi: 10.1016/0038-0717(87)90052-6

[B45] Wang J. Huang J. Zhao X. Wu P. Horwath W. R. LiI H. . (2016). Simulated study on effects of ground managements on soil water and available nutrients in jujube orchards. Land Degrad Dev. 27 (1), 35–42. doi: 10.1002/ldr.2334

[B46] Wang Y. Z. Li Liu L. Yu Luo Y. Mukesh K. A. Yang J. F. Duan Y. M. . (2020). Mulching practices alter the bacterial-fungal community and network in favor of soil quality in a semiarid orchard system. Sci. Total Environ. 725, 138527. doi: 10.1016/j.scitotenv.2020.138527 32304971

[B47] Wang J. Zhang L. H. He X. H. Zhang Y. Q. Wan Y. Duan S. Y. . (2021). Environmental mitigation potential by improved nutrient managements in pear (Pyrus pyrifolia l.) orchards based on life cycle assessment: A case study in the north China plain. J. Clean. Prod. 262, 121273–121282. doi: 10.1016/j.jclepro.2020.121273

[B48] Wardle D. A. Yeates G. W. Bonner K. I. (2001). Impacts of ground vegetation management strategies in a kiwifruit orchard on the composition and functioning of the soil biota. Soil Biol. Biochem. 33 (8), 893–905. doi: 10.1016/S0038-0717(00)00235-2

[B49] Wei H. Xiang Y. Z. Liu Y. Zhang J. E. (2017). Effects of sod cultivation on soil nutrients in orchards across China: a meta-analysis. Soil Tillage Res. 169, 16–24. doi: 10.1016/j.still.2017.01.009

[B50] Xiong W. Li R. Ren Y. Liu C. Zhao Q. Y. Wu H. S. . (2017). Distinct roles for soil fungal and bacterial communities associated with the suppression of vanilla fusarium wilt disease. Soil Biol. Biochem. 107, 198–207. doi: 10.1016/j.soilbio.2017.01.010

[B51] Ye G. Lin Y. Luo J. Di H. J. Lindsey S. Liu D. . (2020). Responses of soil fungal diversity and community composition to long-term fertilization: Field experiment in an acidic ultisol and literature synthesis. Appl. Soil Ecol. 145, 103305. doi: 10.1016/j.apsoil.2019.06.008

[B52] Zhang Q. (1997). A preliminary observation on the vertical distribution of the roots of pear trees. J. Tarim Agric. Univ. 1, 54–556.

[B53] Zhang Q. Pang X. M. Chen X. M. Ye J. H. Lin S. X. Jia X. L. (2020). Rain-shelter cultivation influence rhizosphere bacterial community structure in pear and its relationship with fruit quality of pear and soil chemical properties. Scientia. Horticult. 269, 109419. doi: 10.1016/j.scienta.2020.109419

[B54] Zhao B. X. Yan J. F. Pan F. R. (2022). Effects of grasses on soil microecology in cherry orchards. Southwest. Agric. J. 1–9. doi: 10.16213/j.cnki.scjas.2022.4.020

[B55] Zheng W. Gong Q. Zhao Z. Liu J. Zhai B. Wang Z. (2018). Changes in the soil bacterial community structure and enzyme activities after intercrop mulch with cover crop for eight years in an orchard. Eur. J. Soil Biol. 86, 34–41. doi: 10.1016/j.ejsobi.2018.01.009

[B56] Zhong Y. Yan W. Wang R. Wang W. Shangguan Z. (2018). Decreased occurrence of carbon cycle functions in microbial communities along with long-term secondary succession. Soil Biol. Biochem. 123, 207–217. doi: 10.1016/j.soilbio.2018.05.017

