# Supplementary material for: Effect of planting and mowing cover crops as livestock feed on soil quality and pear production
Source: Front Plant Sci. 2023 Jan 4;13:1105308. doi: 10.3389/fpls.2022.1105308 (PMC9845916; doi:10.3389/fpls.2022.1105308)
Supplement: Supplementary file 1 [file Table_1.docx]

**Table S1**

Effect of different management practices on alpha diversity of soil bacterial and fungal community under different soil layer (0-10cm, 10-20cm and 20-40cm). Values in the same column following different letters suggest significant differences (p < 0.05). Mean ± standard deviation is presented on different treatments. NG, natural grass; MF, planting ryegrass and mowing as livestock feed.

|  | Bacterial | | Fungi | |
| --- | --- | --- | --- | --- |
| Treatment | chao | shannon | chao | Shannon |
| NG 0-10cm | 4549.4±483.4a | 10.0±0.4a | 11.5±2.9a | 2.2±0.3ab |
| NG 10-20cm | 4366.3±745.1a | 9.6±0.7a | 10.0±1.7a | 2.0±0.2ab |
| NG 20-40cm | 4563.0±504.1a | 9.7±0.6a | 10.3±3.1a | 2.2±0.2a |
| MF 0-10cm | 4703.2±530.5a | 10.2±0.4a | 15.1±8.6a | 1.8±0.4b |
| MF 10-20cm | 4649.9±445.9a | 10.1±0.4a | 17.6±10.0a | 1.8±0.4b |
| MF 20-40cm | 4674.3±453.0a | 10.0±0.4a | 11.4±4.0a | 2.2±0.2ab |

**Table S2**

Network of co-occurring bacterial and fungaloperational taxonomic units (OTUs) under different treatments. Only Pearson's correlation coefficient (|r| > 0.8 and p < 0.05) is shown. NG, natural grass; MF, planting ryegrass and mowing as livestock feed.

| Treatment | Nodes | edges | Positive correlation | Modularity | Average degree | Average weight degree | Average path length |
| --- | --- | --- | --- | --- | --- | --- | --- |
| NG 0-10cm | 599 | 14566 | 54.08 | 5.024 | 48.634 | 13.13 | 3.177 |
| NG 10-20cm | 598 | 16200 | 56.36 | 4.238 | 54.181 | 14.528 | 3.128 |
| NG 20-40cm | 600 | 12794 | 62.16 | 1.78 | 42.647 | 23.486 | 3.286 |
| MF 0-10cm | 600 | 13184 | 52.27 | 7.873 | 46.047 | 2.969 | 3.182 |
| MF 10-20cm | 598 | 12831 | 54.48 | 5.205 | 42.913 | 4.184 | 3.213 |
| MF 20-40cm | 600 | 13676 | 53.83 | 5.035 | 45.587 | 3.814 | 3.209 |
